# 

**Published:** 1880-03

**Authors:** 


### CONTENTS:

ARTICLE I.—Extirpation of the Dental Pulp, and Root Fillling. By L. C. Ingersoll, D. D. S. .... 481
ARTICLE II.—Reaction of Oral Fluids. By J. L. Asay, M. D. .... 493
ARTICLE III.—Therapeutical Action of Cold. By W. H. Thomsen, M. D. .... 498
ARTICLE IV.—An Odontome. By G. V. Black, D. D. S. .... 504
ARTICLE V.—The Audiphone and Dentiphone .... 509
ARTICLE VI.—Chloral Inebriety. By Dr. J. B. Mattison .... 512
ARTICLE VII.—A Remarkable Case .... 515
ARTICLE VIII.—Effects of Long-continued Use of Chloral .... 517
ARTICLE IX.—New York Odontological Society .... 519

### EDITORIAL, ETC.

Fortieth Annual Commencement of the Baltimore College of Dental Surgery .... 520
Alumni Meeting, Baltimore College of Dental Surgery .... 523

### MONTHLY SUMMARY.

Duplicating Vulcanite Plates .... 524

Skin—A Microscopical Examination of its Inflammation .... 525

Uralium, a New Metal .... 525

Glycerine Ointment .... 526

Replanted Teeth .... 526

Another Discoverer of Anæsthesia .... 526

The Physiology of Saliva .... 527

Pathological Changes in the Eyes. .... 527

Chloral an Antidote to Strychnine .... 527

Replanting of Teeth .... 528

The Audiphone .... 528

Ink on the Carpet .... 528

Japanese on Blood Letting .... 528

Frequency of Bright's Disease .... 528

